# Relationship between *vacA* Types and Development of Gastroduodenal Diseases

**DOI:** 10.3390/toxins8060182

**Published:** 2016-06-09

**Authors:** Tran Thi Huyen Trang, Tran Thanh Binh, Yoshio Yamaoka

**Affiliations:** 1 Department of Environmental and Preventive Medicine, Oita University Faculty of Medicine, 1-1 Idaigaoka, Hasama-Machi, Yufu-City, Oita 879-5593, Japan; huyentrang110@yahoo.com (T.T.H.T.); binh79@oita-u.ac.jp (T.T.B.); 2 Department of Molecular Biology, 108 Hospital, Hanoi, Vietnam; 3 Department of Endoscopy, Cho Ray Hospital, Ho Chi Minh, Vietnam; 4 Department of Medicine-Gastroenterology, Michael E. DeBakey Veterans Affairs Medical Center and Baylor College of Medicine, Houston, TX 77030, USA

**Keywords:** VacA genotypes, vacuolation, prolonged *Helicobacter pylori* infection, *H. pylori*-mediated diseases

## Abstract

The *Helicobacter pylori* vacuolating cytotoxin (VacA) is a secreted pore-forming toxin and a major virulence factor in the pathogenesis of *H. pylori* infection. While VacA is present in almost all strains, only some forms are toxigenic and pathogenic. While *vacA* and its genotypes are considered as markers of *H. pylori*-related diseases or disorders, the pathophysiological mechanisms of VacA and its genotypes remain controversial. This review outlines key findings of publications regarding *vacA* with emphasis on the relationship between *vacA* genotypes and the development of human disease.

## 1. VacA and Allelic Diversity of *vacA*

*Helicobacter pylori* (*H. pylori*) infection has been implicated in a range of disorders of the upper gastrointestinal tract and associated organs. However, virulence mechanisms responsible for *H. pylori* persistence and clinical outcomes remain controversial. Ongoing efforts to decipher the pathophysiology of *H. pylori* infection have illuminated crucially important contributions of a number of bacterial factors in *H. pylori* pathogenesis. The vacuolating cytotoxin (VacA) has been identified as one of the most important of these virulence factors. As an intracellular-acting protein exotoxin, VacA affects multiple cellular pathways in different host cell types and induces host cell vacuolation and cell death. Furthermore, specific *vacA* genotypes have also been reported to be useful for predicting risk of different clinical outcomes [1,2,3].

**Figure 1:**
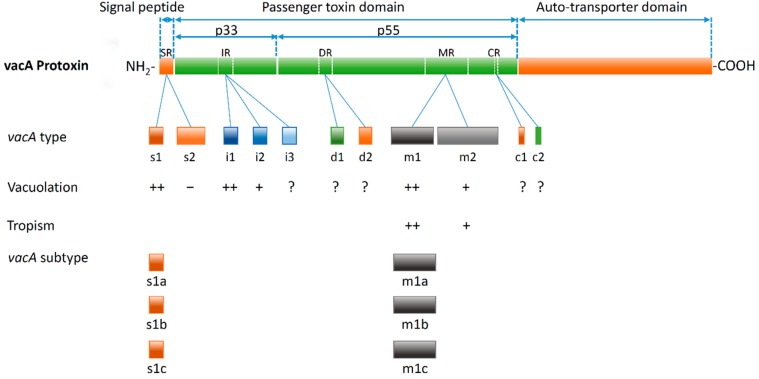
Sequence diversity regions of the *vacA* closely associated with vacuolating activity of *H. pylori* and clinical outcomes are localized to the signal region (SR); the intermediate region (IR) on p33 domain; the d-region (DR), middle region (MR) and c-region (CR) on p55 domain. The different types of these regions are associated with differences in vacuolation, specificity and clinical outcome. The s1, m1, i1 type have been classified as fully active VacA and are associated with a higher risk of development of GC than the s2, m2, or i2. In contrast to the s1 type, the s2 forms of VacA consistently lack detectable vacuolation activity in most *in vitro* assays. In comparison to the m1/i1 types, the m2/i2 types are considerably less active and are virtually nontoxic. The function of the i3 remains undefined. The d-region has been considered to be related with VacA binding to the host gastric cells and vacuolating activity, however, compelling evidence to support this is still lacking. The function of the c-region remains a mystery; however, the c1 genotype has been strongly associated with the risk of GC. The s1 and m1 genotype have been further classified into the three subtypes s1a, s1b, s1c and m1a, m1b, m1c, respectively.

Although nearly all strains isolated from humans possess the *vacA* gene, the capacity to induce cell vacuolization differs significantly from strain to strain [4,5]. Previously, vacuolating cytotoxin phenotypes were determined based on the levels of *vacA* transcription or by the efficiency of VacA secretion [6]. However, subsequent studies have demonstrated that phenotypes are modulated by genetic variation within the *vacA* gene. The three high sequence diversity regions of the *vacA* closely associated with vacuolating activity and with clinical outcomes include the signal (s)-, intermediate (i)-, and middle (m)- region [5,7,8,9,10,11]. The *vacA* s- and the m-regions are the two main polymorphic regions and have been well-characterized as markers of *H. pylori*’s virulence and risk of progression to serious diseases [5,7,12,13,14]. The first polymorphic region of *vacA* (s-region) includes both signal peptides and is defined as a part of the apparatus to transport the mature virulent 88 kDa VacA toxin and the amino-terminal residues of p33 which is predicted as a highly hydrophobic domain of VacA. In contrast, the m-region encodes part of the carboxyl-terminal p55 subunit which has an important role in mediating VacA binding to host cells [15]. The i-region is the third polymorphic determinant and is located in the carboxyl-terminal half of p33 between the s-region and m-region [10]. The i-region appears to interact with the s and m regions and has been described as the determinant of VacA toxicity. Furthermore, the i-region has been suggested to be a better predictor of disease severity than either the s- or m-regions [10,16]. Based on the amino acid sequence differences within these regions, two or more primary variants have been described for each region: s1 and s2 for the signal region; i1, i2 and i3 for the intermediate region; and m1 and m2 for the middle region. Furthermore, the s1 and m1 genotypes have been further classified into three subtypes s1a, s1b, s1c and m1a, m1b, m1c, respectively [5,9,11,17,18,19]. Recently, two additional regions of variation were found in *vacA*: the deletion (d)-region, located between the i- and the m-region exhibiting either d1 genotype without the 69–81 base pair (bp) deletion or d2 genotype with the deletion; and c-region [20,21]. The last includes a deletion of 15 bp located at the 3′-end region sequences of the *vacA* and divided into c1 (with deletion) and c1 (without deletion) (Figure 1).

## 2. The Association of *vacA* Genotypes and Cytotoxin

The most distinct effect of VacA is vacuolation of cells. After VacA internalization, internal membranous vesicles accumulate. Anion-selective channels are created in the membrane of these vesicles which facilitate the transport of chloride ions. VacA induces the accumulation of membrane permeable weak bases and finally results in osmotic swelling and vacuolation [22,23]. Even though the physiological role of vacuolation is still unclear, vacuolation occurring during *H. pylori* infection is thought to disrupt protein trafficking pathways to and from the plasma membrane and consequently affect a number of cellular functions [24,25,26].

*vacA* genotypes rather than simply the presence or absence of VacA have been identified as critical determinants of pathogenesis, Recent studies have explored the contributions of different *vacA* genotypes in relation to vacuolating activity as well as to *H. pylori* pathogenesis. *vacA* s1 encodes a protein which fully exhibits vacuolating activity. *vacA* s2 encodes a protein with a different signal peptide cleavage site, resulting in a 12-amino-acid amino-terminal extension that inhibits vacuolation [27]. Differences in the cell-binding properties of m1 and m2 VacA proteins have also been recognized. For example, the protein encoded by *vacA* m1 causes vacuolation in a wider range of cells than the m2-encoded protein [5,12,19,28]. Many studies have reported the genotype of *vacA* i-region is a marker of disease outcome; however, there has been relatively few studies comparing the activities of the proteins encoded by different types of i-region. *vacA* i1 shows the strongest vacuolating activity on mammalian cells and is thought to cause significantly greater gastric mucosal inflammation compared to infection with i2 containing strains [10,16]. Being phylogenetically closer to the i2 genotype, the i3 genotype is non-vacuolating and has rarely been found [11].

The combination of the *vacA* s-, m-, i-, region genotypes among *H. pylori* strains provides better differentiation of vacuolating activity between strains and clinical outcomes. Variations in the s- and m-regions gives rise to four different *H. pylori* genotypes; s1m1, s1m2, s2m1 and s2m2 with different abilities to induce the formation of acidic vacuoles in infected cells. In general, s1m1 strains produce a large amount of toxin and cause the greatest vacuolization of epithelial cells. In contrast, s1m2 strains may or may not induce cell vacuolation depending on the cell line; most s2m2 strains produce little or no cytotoxin. s2m1 strains are rare and non-vacuolating [6,10,18,29,30]. All s1m1i1 strains are vacuolating and all s2m2i2 are non-vacuolating. s1m2 strains containing the i1 genotype induce cell induce cellular vacuolation while those containing the i2 genotype do not. Thus, s1m1i1 and s1m2i1strains are more virulent and more likely associated with gastric cancer (GC) than the s2m2i2 and s1m2i2 strains [1,10,31].

Although the d1- and c1- genotype have been considered as biomarkers of a strong risk of GC, the contribution of the d- and c-region and their genotypes to cellular vacuolation is as yet undefined. Considering genotypes of the d- and c-region in combination with genotypes of other variant regions, d1/c1 strains are almost exclusively associated with types producing vacuolating cytotoxin (s1/m1/i1) and d2/c2 with non-vacuolating types (s2/m2/i2) [20,21]. Further studies will be required to systematically analyze the role of these regions and their genotypes in the induction of vacuolation.

## 3. The Relationship between *vacA* Polymorphism and *H. pylori* Persistence

After colonizing host cells, the bacteria produce toxins which can cause a broad spectrum of cellular alterations. The toxin has also been hypothesized to enable bacterial colonization by creating extensive remodeling of host cells to provide a more suitable colonization niche. Thus, toxin-producing bacteria may have a selective advantage for colonization compared with strains that do not produce a particular toxin [23]. Since all *H. pylori* strains possess *vacA* and genotype of the *vacA* is significantly associated with the risk of developing a clinical diseases, the secreted pore-forming toxin VacA is thought to facilitate *H. pylori* persistence. It has been proposed that VacA enhances the ability of *H. pylori* to colonize the mouse stomach [32]. However, *H. pylori* strains with *vacA* type s1 and s2 have both been able to colonize the mouse stomach without a significant difference [33]. This has been further clarified by experiments in which it was shown that the expression of the s1i1 or s1i2 genotypes significantly reduced both the proportion of successful infections and gastric colonization density in successfully infected mice compared with the expression of the s2i2 genotype [34]. Other data suggested a correlation between *vacA* genotype with *H. pylori* persistence and antimicrobial susceptibility. For example, most *H. pylori* isolates found to be resistant to all tested antibiotics were positive for the *vacA* s1a m1 genotype [35]. The contribution of the *vacA* genotype to persistent infection has also been related to VacA associated inhibition of the proliferation and immune response of T cells [36]. During *H. pylori* infection, T cells are hypo-responsive thought to be related to the activity of transforming growth factor β (TGF-β) exerting a suppressive effect on T cells. The expression levels of mucosal TGF-β1 have been demonstrated to be dependent on the *vacA* genotypes, with a positive correlation being reported between secreted *vacA* s1 or s1m1 types and increased mucosal TGF-β1 mRNA levels. Thus, the *vacA* s1m1 genotypes have been thought to contribute to persistent infection with *H. pylori*. On the other hand, the role of the active genotype s1 in *H. pylori* persistence has been suggested to be related to preserving the niche for *H. pylori* by inducing attenuation of the tumor necrosis factor-related apoptosis-inducing ligand (TRAIL) system [37]. The down-regulation of the TRAIL system, in the context of an *H. pylori* infection, may limit apoptosis of gastric epithelial cells and destruction of tissues necessary for *H. pylori* to maintain its niche and survival in gastric epithelial cells. The down-regulation of the TRAIL system has been shown to be reduced in patients infected with *H. pylori* carrying the *vacA* s1 compared to those carrying the *vacA* s2. Prolonged *H. pylori* infection has been presumed to lead to saturation of cellular repair capabilities, the loss of glands, resulting in atrophic gastritis, followed by the development of intestinal metaplasia and dysplasia [38,39,40]. Thus, by facilitating *H. pylori* consistent colonization in the host cells, active VacA producing strains may contribute to *H. pylori* disease pathogenesis.

## 4. Role of the *vacA* Genotype in Gastroduodenal Diseases

*H. pylori* infection increase apoptosis in the gastric mucosa which may contribute to gastric diseases including peptic ulcer diseases (PUD) and GC [15,41,42,43,44]. In an effort to characterize the role of different *vacA* genotypes in *H. pylori*-associated gastroduodenal diseases, the effect of the *vacA* genotypes on gastric epithelial cells has been increasingly investigated. Numerous studies have provided evidence for a stronger association of disease outcomes in individuals infected with *H. pylori* strains possessing active *vacA* genotypes such as s1, m1, i1, d1 than that with less or non-active *vacA* types [5,14,15,18]. The prevalence of these genotypes in patients with PUD and GC is significantly greater than among those with gastritis alone [2,14,45]. The differential geographic distribution of these genotypes has also suggested the prevalence of *vacA* types was higher in areas where the incidence of GC is high compared to areas with a low incidence of GC [46].

An association of *vacA* s1 strains and enhanced gastric mucosal inflammation was found in very early studies evaluating the contribution of the *vacA* genotype in gastroduodenal diseases [30,47,48]. The *vacA* s1 allele was specified to be correlated with extensive chronic inflammation independently of the *cagA* status, possibly through its enhanced ability to produce the vacuolating cytotoxin [48]. The *vacA* s1 genotype has been associated with the higher levels of interleukin (IL)-8 in the gastric mucosa, chronic inflammation, neutrophil activity, epithelial damage; and with atrophy and intestinal metaplasia rather than the s2 genotype [16,39,49,50]. Thus, strains harboring the s1 allele have been considered to be associated with an increased risk of gastric carcinoma than infection with s2 allele containing strains.

*vacA* m1 strains have also been associated with gastric diseases and GC compared to m2 strains. The m1 genotype has proven to be a better marker than *cagA* status or *vacA* s1 genotype for describing the difference of the incidence of GC among East Asian countries where nearly all strains are East Asian types *cagA* and *vacA* s1 [46]. However, some studies have indicated that *vacA* m-region typing alone is insufficient to distinguish gastroduodenal diseases or identify an increased incidence of GC [49,51]. When m-region genotypes are combined with other *vacA* genotypes or other virulent factors such as *cagA*, the contributions of the *vacA* genotypes to lesion progression of infected patients are more evident [52,53]. The prevalence of *vacA* s1m1 and s1m2 genotypes differ among intestinal GC (IGC) and diffuse GC (DGC) suggesting that these genotypes may play different roles in the pathogenesis of IGC and diffuse DGC [54]. The s1m1 genotype has been found more frequently in children with ulcers, whereas the s2/m2 genotype has been more frequent in patients with gastritis and gastroesophageal reflux disease (GERD) [55]. *cagA*-positive strains also correlated with severe histopathological damage and CagA is most commonly associated with the *vacA* s1 genotype, particularly in patients with PUD or GC [56]. Additionally, the *cagA*+ *vacA* s1 genotye is present in patients with high expression of tumor necrosis factor receptor-associated factor 1 (TRAF1), tumor necrosis factor receptor superfamily member 9 (4-1BB), or B-cell lymphoma-extra-large (Bcl-xl) [57]. The expression of TRAF1, and Bcl-xl in human gastric epithelial cells is significantly upregulated in intestinal metaplasia and gastric carcinoma patients whereas in atrophic gastritis there is low expression of these factors. Overall, it seems likely that *cagA*+/*vacA* s1m1 strains upregulate TRAF1 activation, which triggers 4-4BB mediated Bcl-xL activation, thereby exerting an antiapoptotic effect and contributing to the pathogenesis of GC [57]. Interestingly, one study reported a trend for more virulent *cagA*+/*vacA* s1m1 strains to be present in younger individuals [49], suggesting that the virulence of the infecting strain in early life may determine outcome.

The contribution of genotypes of i-region to gastric diseases has been varied among epidemiology studies [10,16,58,59]. However, most studies confirm tht *vacA*i genotypes are better predictors of risk of GC or duodenal ulcer than other *vacA* alleles, *cagA* status, or the size of the *cagA* 3′ variable repeat region [2,60,61]. The strong correlation between the *vacA* i1 strains and PUD has been recently highlighted [21], and no significant correlation was found between genotypes of s-, m-, d- or c-region, whether independently or in combination and PUD as only the i1 type was linked to an increased risk of PUD [21]. The authors suggested that the carriage of both the i1 type and *cagA* further increased the risk of PUD in an Iranian population. The ability of the *vacA*i region to identify patients at higher risk of GC development may be an increased capacity to inhibit activation of nuclear factor of activated T cells (NFAT) and suppress IL-2 production [62]. A comparison of the ability of the i1 and i2 genotypes to cause functional alterations in Jurkat cells did not reveal differences in the capacity of the i1 and i2 forms in causing vacuolation of RK13 cells; however, i2 forms bound to Jurkat cells less avidly than the i1 forms; i2 forms had a diminished capacity to inhibit the activation of NFAT and suppress IL-2 production compared to the i1 form. Regarding the combination of 3 *vacA* regions (s, m, i), the authors found that only naturally occurring s1m2 strains varied in i-types with s1m1 and s2m2 strains were exclusively i1 and i2, respectively.

Even though knowledge of the structure–function relationships of the *vacA* d region is limited, *vacA* d1 strains have been proposed as a new determinant of GC risk and for the potential for atrophy compared to the s-, m-, and i-regions [20,63]. Moreover, s1 strains with the d1 genotype were also considered as risk biomarkers in areas of high GC incidence in Iran [64]. Although the biologic role of the c-region in vacuole-creating activities is yet to be understood, the *vacA* c1 type has been strongly associated with the risk of GC [21]. Furthermore, the combination between the c1 type with *vacA*-m1, -i1, -d1 and *cagA*+ showed a further increase in susceptibility to GC; the combination of c1/i1 type showed the highest risk of GC.

The *vacA* genotypes have been demonstrated to be better determinants of the severity of gastric damage compared to the bacterial load. Winter *et al.* compared *vacA* genotypes associated-gastric damage in infected-humans and -mice [34] and, as discussed above, compared with the less-active *vacA* s2i2 strains, strains producing the s1i1 and the s1i2 showed reduced colonization rates and bacterial densities. However, no significant correlation between colonization density and gastric pathology was observed. Despite colonizing poorly, the s1i1 strains consistently induced more severe and extensive inflammation and spasmolytic polypeptide expressing metaplasia (SPEM) than the s2i2 strains. The finding supported the hypothesis that the induction of inflammation and severe gastroduodenal diseases more related to VacA expression than bacterial density. This finding is in agreement with previous study which showed *H. pylori* genotype is more relevant than bacterial density for induction of oxidative DNA damage involved in gastric carcinogenesis [65]. In that study, density of *H. pylori* was not associated with the level of oxidative DNA damage whereas the more-virulent s1 and m1 strains were associated with higher levels of oxidative DNA damage than the s2 and the m2 containing strains, respectively.

## 5. Role of *vacA* Genotype in Extragastroduodenal Diseases

*H. pylori*-mediated diseases from outside of the gastrointestinal track such as disorder in lung or heart have attracted the attention of researchers. *H. pylori* infection was associated with reduced lung function that is most likely due to the effect of the bacterium on lung growth earlier in life [66]. *H. pylori* infection has also been associated with systemic inflammation and increased risk of cardiovascular mortality in patients with chronic obstructive pulmonary disease (COPD) [66]. Nevertheless, the attribution of *H. pylori* virulence including the *vacA* genotypes to development of these diseases remains unclear. To test the importance of *H. pylori* presence in the pathophysiology of upper respiratory diseases in children, a study conducted on children undergoing surgery for adenotonsillar hypertrophy reported that 98% of the sample was positive for *H. pylori* with predominance of the s1bm2 genotype [67]. Recently, a study demonstrated for the first time that VacA was present in human lung tissues and was more prevalent in lungs of patients with collagen vascular disease-associated interstitial pneumonia than in those of patients with idiopathic pulmonary fibrosis, nonspecific interstitial pneumonia and cryptogenic organizing pneumonia [68]. The findings of the study suggested that induction of IL-8 and IL-6 in airway epithelial cells had a specific reaction against VacA stimulation. Consistent with this finding, another study on the specific relationship between smoking and bacterial load has noted the evidence of the association of the *vacA* i1 genotype with active-smoking [69]. According to a report of the Surgeon General, the cardiovascular risks were reported to be attributable to active-smoking. Therefore, the correlation of the *vacA* genotypes in development of diseases in *H. pylori* infected smokers needs to be considered in further study. These correlations remain speculative and it is still unclear whether their presence is restricted to *H. pylori*.

## 6. Conclusions

*H. pylori*-associated clinical outcomes relate in part to the genetic diversity among clinical *H. pylori* isolates. Although all *H. pylori* strains possess *vacA*, the secretion and toxicity of VacA are dissimilar among strains. These differences correlate to *vacA* polymorphisms. A number of recent studies have demonstrated clinical and biological differences between strains containing different *vacA* polymorphisms. Detailed studies of the biological activities and clinical associations related to different *vacA* genotypes are required for a deeper understanding of their role in *H. pylori*-mediated diseases and for the development of novel strategies to counter their effects.
